# Safety and efficacy of a nonionic water-soluble iodinated contrast medium for videofluoroscopic swallowing studies

**DOI:** 10.1007/s11282-026-00959-x

**Published:** 2026-08-01

**Authors:** Masaru Konishi, Toshikazu Nagasaki, Kiichi Shimabukuro, Sachiko Yamada, Mineka Yoshikawa, Naoya Kakimoto

**Affiliations:** 1 Department of Oral and Maxillofacial Radiology, Hiroshima University Hospital, 1-2-3 Kasumi, Minami-ku, Hiroshima, 734-8553 Japan; 2 Department of Oral and Maxillofacial Radiology, Graduate School of Biomedical and Health Sciences, Hiroshima University, Hiroshima, Japan; 3 Division of Specific Dentistry, Speech Clinic, Hiroshima University Hospital, Hiroshima, Japan; 4 Department of Advanced Prosthodontics, Graduate School of Biomedical and Health Sciences, Hiroshima University, Hiroshima, Japan

**Keywords:** Dysphagia, Swallowing disorder, Videofluoroscopic swallowing study, Fluoroscopy, Contrast agent

## Abstract

**Objectives:**

This study evaluated the safety and efficacy of an iodinated contrast agent for videofluoroscopic swallowing studies (VFSS) in patients with swallowing disorders, compared with barium sulfate contrast agents.

**Methods:**

The study included 20 patients who underwent VFSS at Hiroshima University Hospital in 2025 for dysphagia evaluation. Iodixanol (Visipaque 270®; GE Healthcare Pharma Co., Ltd., Chicago, Illinois) was used as the iodinated contrast agent. For comparison, VFSS images obtained with Barytester® (FUSHIMI Pharmaceutical Co., Ltd., Kagawa, Japan) in the same patients served as barium sulfate controls. The frequency of adverse events associated with oral Visipaque 270® administration was assessed. VFSS images obtained with Barytester® and Visipaque 270® in the same patients were compared to evaluate imaging performance. To determine whether image evaluation outcomes were equivalent between Visipaque 270® and Barytester®, an equivalence test was performed using the two one-sided tests procedure.

**Results:**

Visipaque 270® was associated with no adverse events, including gastrointestinal or other adverse events. Image evaluations using Visipaque 270® and Barytester® were statistically equivalent.

**Conclusions:**

VFSS was performed using the iodinated contrast agent Visipaque 270®. No adverse events related to oral Visipaque 270® administration were observed, and contrast enhancement on VFSS images was comparable to that achieved with Barytester®.

**Trial registration:**

Japan Registry of Clinical Trials, jRCTs061240040; registered 26 July, 2024.

## Introduction

The primary causes of dysphagia include cerebrovascular disorders, neuromuscular diseases, dementia, and head and neck cancer. In aging populations, the number of patients with dysphagia is increasing [1–3]. Patients with mastication and swallowing disorders face complications such as aspiration pneumonia and malnutrition due to reduced food intake and insufficient essential nutrient intake [4–6]. Therefore, accurately diagnosing mastication and swallowing disorders is essential for preventing aspiration and malnutrition.

Videofluoroscopic swallowing study (VFSS) is the standard diagnostic method for mastication and swallowing disorders during the oral and pharyngeal phases. Barium sulfate, which is indicated for gastrointestinal contrast examinations, has been widely used since the introduction of VFSS [7]. In VFSS, barium sulfate agents indicated for gastrointestinal fluoroscopy are routinely used. These agents are suspended in water and used as liquids or added to food in the testing procedure. However, reports indicate that aspirated barium sulfate can remain in the lungs for a prolonged period and form granulation tissue [8]. Clinically, a fatal case due to aspiration has been reported after the administration of large volumes of concentrated solutions [9]. Therefore, barium sulfate is not recommended for VFSS in patients at high risk of aspiration. However, currently available contrast agents suitable for VFSS are limited to barium sulfate.

This study focused on nonionic water-soluble iodinated contrast agents, commonly used in computed tomography scans and other procedures as alternatives to barium sulfate preparations. Reports indicate that these agents did not induce pulmonary inflammatory reactions following aspiration experiments in rats [10]. Consequently, they are considered relatively safe contrast agents, even if aspirated. Furthermore, because nonionic water-soluble iodinated contrast agents are colorless, transparent, and odorless liquids, adding them to food causes almost no changes in appearance, texture, or flavor, making them potentially useful contrast agents for VFSS. However, most nonionic water-soluble iodinated contrast agents are not approved for oral use and are not permitted for VFSS.

The use of nonionic water-soluble iodinated contrast agents in VFSS may provide greater contrast enhancement than barium sulfate and enable safer examinations with fewer adverse effects. Applying these agents to test foods may improve the texture of swallowing test foods containing contrast media, prevent worsened mastication and swallowing due to poor texture, and enable more accurate swallowing assessments [11].

This study evaluated the safety and efficacy of using a nonionic water-soluble iodinated contrast agent for VFSS in patients with mastication and swallowing disorders, compared with barium sulfate contrast agents.

## Materials and methods

This was a single-arm interventional study conducted at a single center to evaluate the safety and efficacy of a nonionic water-soluble iodinated contrast agent for VFSS. Visipaque 270® (GE Healthcare Pharma Co., Ltd., Chicago, Illinois), which contains iodixanol as its main component, was used as the nonionic water-soluble iodinated contrast agent. VFSS images obtained with barium sulfate (Barytester®, FUSHIMI Pharmaceutical Co., Ltd., Kagawa, Japan) in the same patients were used as controls.

### Patients

This study included 20 patients who underwent VFSS at Hiroshima University Hospital in 2025 for dysphagia evaluation. These patients met all inclusion and exclusion criteria. The characteristics of the 20 patients included in this study are shown in Table 1. The cohort comprised 15 male and five female patients, with a mean age of 69.1 years. All patients had previously undergone contrast-enhanced computed tomography (CT) using an iodinated contrast agent. The median interval between VFSS examinations using Barytester® and Visipaque 270® was 35 days (range: 17–1407 days).

**Table 1:** Patient characteristics

Case number	Age	Sex	Primary lesion	Neck dissection	Radiation therapy	Experience of contrast-enhanced CT with iodinated contrast agents	Timing of VFSS with Barytester®	Timing of VFSS with Visipaque 270®	Intervals between VFSS with Barytester®and Visipaque 270® (days)
1	79	M	Buccal mucosa cancer	+	+	+	Before surgery	After surgery	1407
2	64	M	Tongue cancer	+	–	+	Before surgery	After surgery	42
3	72	M	Tongue cancer	+	–	+	Before surgery	After surgery	21
4	77	F	Upper gingival cancer	–	–	+	Before surgery	After surgery	21
5	66	M	Lower gingival cancer	+	–	+	Before surgery	After surgery	21
6	75	M	Upper gingival cancer	–	–	+	Before surgery	After surgery	17
7	71	M	Upper gingival cancer	+	+	+	After surgery	After surgery	700
8	76	M	Upper gingival cancer	+	+	+	After surgery	After surgery	364
9	56	M	Lower gingival cancer	+	–	+	Before surgery	After surgery	59
10	53	M	Upper gingival cancer	+	–	+	Before surgery	After surgery	17
11	71	M	Sublingual gland cancer	+	–	+	Before surgery	After surgery	28
12	64	M	Buccal mucosa cancer	+	–	+	Before surgery	After surgery	91
13	76	M	Floor of mouth cancer	+	–	+	After surgery	After surgery	213
14	72	F	Buccal mucosa cancer	+	–	+	Before surgery	After surgery	35
15	71	F	Upper gingival cancer	+	-	+	Before surgery	After surgery	35
16	65	M	Lower gingival cancer	+	–	+	Before surgery	After surgery	73
17	58	F	Lower gingival cancer	+	–	+	Before surgery	After surgery	32
18	68	M	Upper gingival cancer and mandibular osteoradionecrosis	+	+	+	Before surgery	After surgery	21
19	69	M	Floor of mouth cancer	+	–	+	Before surgery	After surgery	35
20	78	F	Lower gingival cancer	+	–	+	Before surgery	After surgery	21

*M* male, *F* female, + present/yes, – absent/no, *CT* computed tomography, *VFSS* videofluoroscopic swallowing study

Inclusion criteria:

1. Patients who had previously undergone VFSS with barium sulfate at Hiroshima University Hospital.
2. Patients with dysphagia who are at least 18 years of age at the time of informed consent.
3. Patients with dysphagia who are eligible for adverse-event assessment until 1 month after VFSS.
4. Patients with dysphagia who provided written informed consent to participate in this study.

Exclusion criteria:

1. Patients with iodine allergy.
2. Patients with severe thyroid disease.
3. Patients with serious renal disease.
4. Patients with serious cardiac disorders.
5. Patients with macroglobulinemia.
6. Patients with multiple myeloma.
7. Patients with tetany.
8. Patients with or suspected of having pheochromocytoma.
9. Patients with a personal or family history of bronchial asthma, rash, urticaria, or other allergic reactions.
10. Patients with a history of drug hypersensitivity.
11. Patients with dehydration.
12. Patients with untreated hypertension.
13. Patients with arteriosclerosis.
14. Patients with diabetes mellitus without renal disorders.
15. Patients with acute pancreatitis.
16. Patients with biliary tract infection.
17. Patients with severe hepatic disorder.
18. Patients with a history of gastrointestinal tract disease or surgery.
19. Women who are pregnant or may become pregnant, women who are breastfeeding, or women who wish to become pregnant.
20. Patients who are participating or intend to participate in other clinical studies while participating in this study.
21. Patients whose participation would not be in their best interest or would interfere with protocol-specific evaluation.
22. Any principal investigator or institutional employee directly involved in this or other clinical research, or any family member of such an employee or principal investigator.

### Study procedures

After informed consent was obtained, patients were enrolled, and VFSS using Visipaque 270® as the contrast agent was performed. After the examination, adverse events were assessed from the start of VFSS until 1 month after the examination. Taste evaluation of Visipaque 270® was performed immediately after the examination. The visibility of Visipaque 270® and Barytester® images from previous examinations was evaluated using the respective images.

### VFSS

VFSS was performed using a digital fluorographic system (Ultimax-I, CANON MEDICAL SYSTEMS CORPORATION, Tochigi, Japan), with patients in an upright sitting position at a frame rate of 30 frames/s. Each patient swallowed 3 mL of both barium sulfate and iodinated contrast agent. Barium sulfate was adjusted to a concentration of 30% w/v, and the iodinated contrast agent was diluted twofold.

### Evaluation

1. Adverse events associated with Visipaque 270®

- Gastrointestinal: diarrhea, nausea, vomiting, abdominal pain, abdominal discomfort, dyspepsia, anal pain and bleeding, constipation, renal failure, gastrointestinal perforation, intestinal obstruction, peritonitis.
- Hypersensitivity: itching, urticaria, rash, erythema, fever, shock, anaphylaxis.
- Respiratory: aspiration pneumonia, pulmonary edema.
- Other events: ventricular fibrillation, seizure.

2. Visibility of contrast agents in VFSS and aspiration during VFSS
  For subjective visibility assessment, three dentists experienced in VFSS performed a blinded 5-point assessment of VFSS images from 20 patients. Specifically, VFSS images obtained with Visipaque 270® were rated on a 5-point scale: 1 (very poor), 2 (poor), 3 (adequate), 4 (good), and 5 (excellent). Before assessment, the evaluators standardized their criteria using previously acquired VFSS images obtained with Barytester®. Previously acquired VFSS images obtained with Barytester® in the same patients were used, and image assessments were conducted using the same 5-point scale as for Visipaque 270®. Assessment results for images obtained with Visipaque 270® were compared with those for images obtained with Barytester®. The evaluators were blinded to the contrast agent used, and three evaluators independently assessed the images. To assess agreement among the three dentists (evaluators), the number of cases with agreement and inter-rater agreement were evaluated using the intraclass correlation coefficient (ICC). The mean scores for Visipaque 270® and Barytester®, along with the mean difference between them, were calculated. For each patient, the mean of the three evaluators’ scores was calculated, followed by a paired t-test.
  JMP Pro version 19.0 (SAS Institute, Cary, NC, USA) and SPSS (IBM Corp., Armonk, NY, USA) were used for all statistical analyses. Statistical significance was set at *P* < 0.05. To demonstrate equivalence in image evaluation results between Visipaque 270® and Barytester®, an equivalence test was performed using two one-sided tests (TOST) with a 5% relative margin [12]. TOST examines the null hypotheses that the true mean difference lies outside the prespecified equivalence margins. If both null hypotheses are rejected, the true mean difference is considered to fall within the prespecified equivalence margins, indicating equivalence between the image evaluation results for Visipaque 270® and Barytester®. A significance level of α = 0.05 was applied. The three dentists independently reviewed VFSS images and assessed the presence of aspiration. Aspiration was diagnosed if any one of the three dentists identified aspiration.
(3) Taste evaluation
  Taste assessment: The taste of Visipaque 270® was rated on a 5-point scale: 1 (very bad), 2 (bad), 3 (adequate), 4 (good), and 5 (very good). Taste assessment had not been performed for Barytester®; historical data was used for the comparisons.

### Ethical considerations

This study was approved by the Hiroshima University Ethics Committee (approval number CRB2024-0002) and was conducted in accordance with the ethical standards of the institutional and/or national research committee and with the 1964 Declaration of Helsinki and its later amendments or comparable ethical standards. The study was also registered with the Japan Registry of Clinical Trials (jRCTs061240040). Written informed consent was obtained from all patients.

## Results

**Fig. 1:**
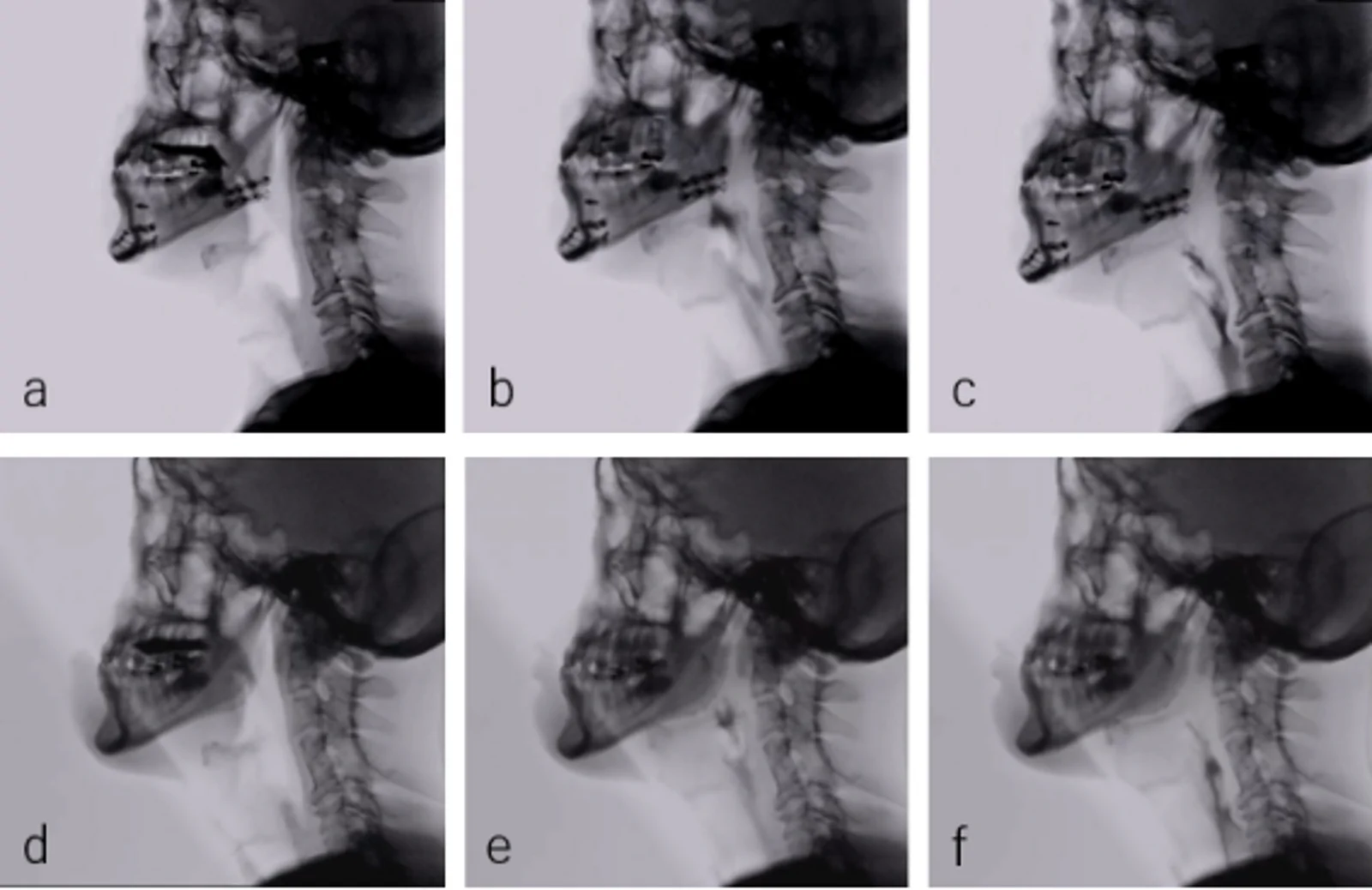
Examples of 5-point VFSS image evaluation. **a** Oral phase with Visipaque 270®. **b** Pharyngeal phase with Visipaque 270®. **c** Pharyngeal and esophageal phase with Visipaque 270®. **d** Oral phase with Barytester®. **e** Pharyngeal phase with Barytester®. **f** Pharyngeal and esophageal phase with Barytester®. For VFSS images obtained with Visipaque 270® (**a**–**c**), all three evaluators rated them 5 (excellent). For VFSS images obtained with Barytester® (**d**–**f**), two evaluators rated them 4 (good), and one evaluator rated them 3 (adequate)

1. Adverse events
  Visipaque 270® caused no adverse events, including gastrointestinal or other adverse effects.
2. Visibility and aspiration assessment of VFSS images among the three dentists
  An example of the three evaluators’ ratings of VFSS image visibility is shown in Fig. 1. Figure 1a–c show VFSS images obtained with Visipaque 270®, which all three evaluators rated as 5 (excellent) on a 5-point scale. Figure 1d–f show images obtained with Barytester®, with two of the three evaluators rating them as 4 (good) and one rating them as 3 (adequate).

With Visipaque®, 14 out of 20 cases showed complete agreement among all three evaluators; five cases showed agreement between two evaluators; and one case showed complete disagreement among all three evaluators. With Barytester®, six out of 20 cases showed complete agreement among all three evaluators; 13 cases showed agreement between two evaluators; and one case showed complete disagreement among all three evaluators. ICCs of the evaluation of three evaluators with Visipaque® and Barytester® were 0.402 (95%CI 0.285–0.747) and 0.473 (95%CI 0.09–0.760).

**Fig. 2:**
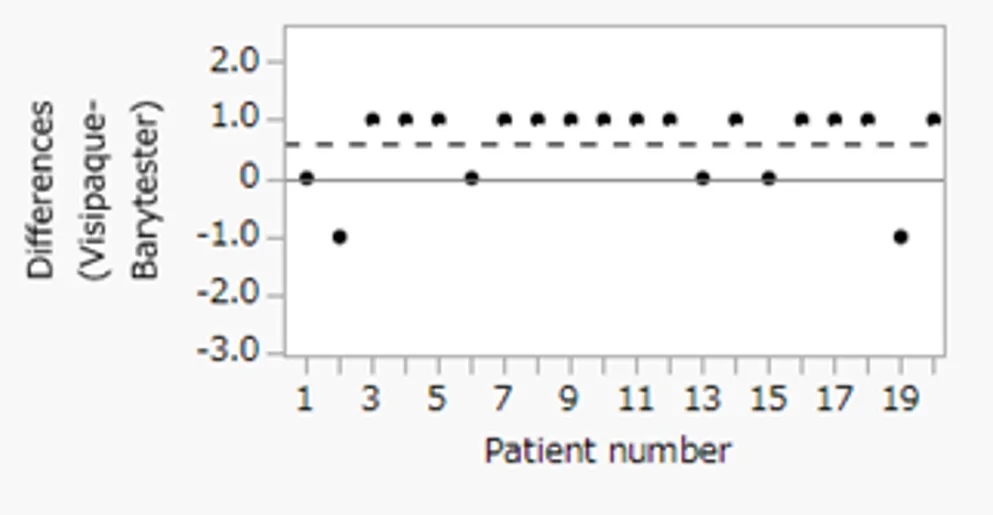
Differences between Visipaque 270® and Barytester® scores. The 5-point image evaluation scale results were a mean ± standard deviation of 4.85 ± 0.40 and 4.53 ± 0.57 for Visipaque 270® and Barytester®, respectively. No statistically significant difference was observed between the scores with the two contrast agents (*P* = 0.997). Horizontal axis: Patient number. Vertical axis: Difference between the Visipaque 270® and Barytester® scores on the 5-point image scale. Dotted line: Mean

**Fig. 3:**
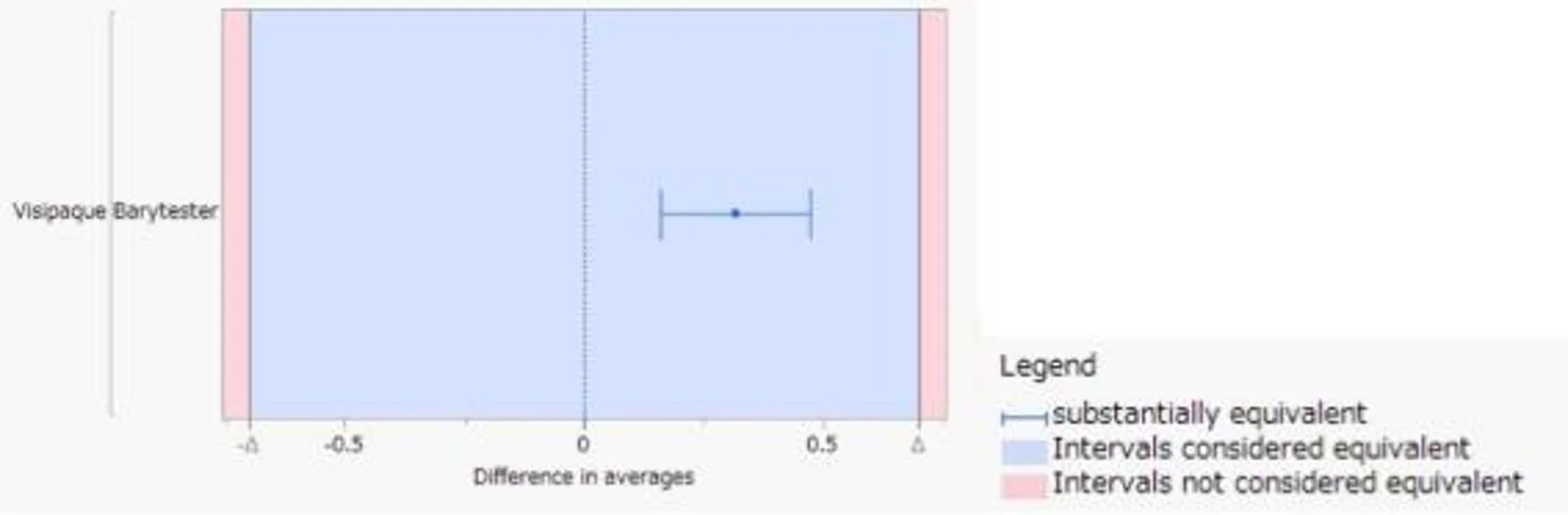
Two one-sided tests (TOST) for demonstrating equivalence. The image evaluation results obtained with Visipaque 270® and Barytester® were substantially equivalent (*P* < 0.0001)

As shown in Fig. 2, scores for each patient were calculated by subtracting the Barytester® score from the Visipaque 270® score. The displayed scores represent the combined evaluation results from the three dentists. The 5-point image evaluation scale results were a mean ± standard deviation of 4.85 ± 0.40 (range: 3–5, median: 5) and 4.53 ± 0.57 (range: 3–5, median: 5) for Visipaque 270® and Barytester®, respectively. No statistically significant difference was observed between the scores with the two contrast agents (*P* = 0.997). Results of the TOST evaluating the equivalence of image assessment between Visipaque 270® and Barytester® are shown in Fig. 3. The image evaluation results obtained with Visipaque 270® and Barytester® were substantially equivalent (*P* < 0.0001).

Eight patients (patient nos. 2, 3, 5, 7, 8, 11, 13, and 16) examined with Visipaque 270® were diagnosed with aspiration by at least one of the three dentists.

3. Taste evaluation
  Taste evaluation for Visipaque 270® showed a mean **±** standard deviation of 3.65 ± 0.74 (range: 2–4, median: 4).

## Discussion

In VFSS, barium sulfate, commonly used in upper gastrointestinal examinations, is typically used as the contrast agent. In this study, an iodinated contrast agent was used for VFSS. The approved indications for Visipaque 270®, the contrast agent used in this study, are cerebral angiography, peripheral vascular angiography, retrograde urogram, and endoscopic retrograde cholangiopancreatography. Oral administration is not approved. Therefore, adverse events after oral administration were a concern. However, in this study, no gastrointestinal, hypersensitivity, respiratory, or other adverse events, including ventricular fibrillation and seizure, were observed after oral administration of Visipaque 270®.

Among iodinated contrast agents, only Gastrografin® (Bayer, Osaka, Japan), an ionic sodium amidotrizoate meglumine solution, is indicated for gastrointestinal tract examination. For contrast agents used in VFSS, essential requirements include safety, taste, and texture. Cost must also be considered. Barium sulfate, if aspirated, has been reported to cause mild to severe complications [8, 9]. Therefore, barium sulfate use for VFSS should be avoided when significant aspiration is clearly anticipated. Among water-soluble iodinated contrast agents, ionic types can cause pulmonary complications, such as neutrophilic exudation, alveolar epithelial damage, congestion, hemorrhage, and edema, if aspirated [13]. Gastrografin®, which is orally administered, is ionic and is therefore not recommended when aspiration risk is present [14, 15]. However, nonionic types have been reported to cause no such complications or only mild complications [8]. Among the 20 patients in this study, eight were diagnosed with aspiration; however, none developed pulmonary complications. This finding is consistent with a report of 53 patients who experienced aspiration during VFSS using a nonionic iodinated contrast agent, with no cases of contrast agent retention in the lung or severe pulmonary complications observed [16]. In addition, no clear signs of inflammation were observed in the lungs, either histologically or through molecular biological analysis, in animal studies involving intratracheal administration of nonionic iodinated contrast agents to rats [10]. Additionally, caution is required regarding hypersensitivity to iodinated contrast agents. All 20 patients in this study had previously undergone CT using iodinated contrast agents for diagnosis of their primary lesions, and no hypersensitivity symptoms were observed during those examinations. No adverse effects due to hypersensitivity to iodinated contrast agents were observed during VFSS. Concerns regarding hypersensitivity are considered low in patients who have previously undergone CT using iodinated contrast agents without adverse reactions.

Regarding taste, barium sulfate is flavored with vanilla essence, giving it a yogurt-like flavor that is generally acceptable. However, because it is supplied as a powder, a powdery taste may remain, resulting in an unpleasant taste sensation. Furthermore, although the yogurt flavor may be acceptable in liquid form, adding it to various foods can sometimes compromise their original taste. Among water-soluble iodinated contrast agents, ionic contrast agents are particularly bitter. Among nonionic agents, monomeric types tend to be rather bitter, whereas dimeric types are less bitter, and some even have strong sweetness. In this study, the taste evaluation for Visipaque 270® showed mean and median scores of 3.65 and 4, respectively, indicating favorable taste results ranging from adequate to good.

A disadvantage of iodinated contrast agents compared with barium sulfate is their substantially higher cost. Iodinated contrast agents used in CT are typically available in 100 mL vials. Each vial is approved for use in only one patient, and once opened, it cannot be stored for later use. The 100 mL full-volume use is difficult in case of VFSS. The remaining contrast agent must be discarded, resulting in a substantial cost burden. Visipaque 270®, used in this study, is supplied in 20 mL vials [17]. This volume is suitable for complete use in a single VFSS. If there is additional requirement during the examination, additional vials can be used individually, minimizing waste [18, 19]. In our previous review of contrast agents used in VFSS, 35 studies reported the use of iodinated contrast agents. The most frequently used agent was Gastrografin® (amidotrizoic acid), followed by Omnipaque® (iohexanol), Gastromiro® (iopamidol), and Isovist® (iotrolan) [20]. Iodinated contrast agents have been reported to exhibit superior permeability and texture compared with barium sulphate when added to various foods during VFSS [18, 19, 21, 22]. Therefore, these factors should be considered when selecting a contrast agent suitable for VFSS.

Although the results regarding image visibility were equivalent for Visipaque® and Barytester®, the inter-rater agreement was moderate, with an ICC of 0.402 for Visipaque® and 0.473 for Barytester®. However, for both Visipaque® and Barytester®, the results of at least two of the three evaluators were consistent in 19 out of 20 cases. Therefore, further improvement can be expected by clarifying and standardizing the evaluation criteria prior to VFSS assessment.

This study has some limitations. First, because data and images from VFSS with Barytester® were retrieved retrospectively from medical records, data on adverse events or taste assessment could not be obtained using the same method as for Visipaque 270®. Furthermore, because the study included patients with oral cancer, all examinations with Visipaque 270® were performed after surgery. In contrast, most VFSS examinations with Barytester® were performed preoperatively, which may have led to substantial differences in swallowing status and influenced image evaluation. And, adverse-event assessment in this study involved collecting patient symptoms during and after VFSS and, for some patients, reviewing blood test results, chest X-rays, and CT images. Some adverse events may therefore have been undetected based on these data alone. Further studies with larger patient cohorts are warranted. Finally, this study aimed to investigate the safety of oral administration of Visipaque®, an iodine-based contrast agent not approved for oral use. In this context, it should be noted that patient enrollment was limited to 20 cases, and the volume of contrast agent administered to patients was planned to be as small as 3 mL. Therefore, due to the small number of cases and small bolus volume, it is possible that image quality could not be adequately evaluated.

In conclusion, VFSS was performed with the iodinated contrast agent Visipaque 270® in this study. No adverse events associated with oral Visipaque 270® administration were observed, and contrast enhancement on VFSS images was equivalent to that observed with the barium sulfate agent Barytester®. Future research will examine the efficacy of Visipaque 270® on VFSS by using higher doses of the contrast agent and by adding it to various foods.

## Data Availability

The data that support the findings of this study are not publicly available because they contain clinical information that could compromise patient privacy but are available from the corresponding author upon reasonable request and with appropriate institutional approval.
